# Effect of fermented dandelion on productive performance, meat quality, immune function, and intestinal microbiota of broiler chickens

**DOI:** 10.1186/s12917-023-03751-9

**Published:** 2023-09-29

**Authors:** Jinju Mao, Yuan Wang, Ting Duan, Na Yin, Chenlin Dong, Xuerong Ren, Na Liu, Xiaoping An, Jingwei Qi

**Affiliations:** 1 College of Animal Science, Inner Mongolia Agricultural University, Hohhot, 010018 China; 2 Inner Mongolia Herbivorous Livestock Feed Engineering Technology Research Center, Hohhot, 010018 China; 3 Key Laboratory of Smart Animal Husbandry at Universities of Inner Mongolia Automomous Region, Hohhot, 010018 China

**Keywords:** Fermented dandelion, Broilers, Production performance, Meat quality, Immune function, Intestinal microbiota

## Abstract

**Background:**

Dandelion has a great potential to be used as feed additive. Using microbial fermentation technology to degrade cell walls is conducive to enable better release of bioactive compounds of dandelion. This study intended to explore the effect of fermented dandelion (FD) on production performance, meat quality, immune function, and intestinal microbiota of broiler chickens. One-hundred and twenty 1-day-old male Arbor Acres broiler chickens were randomly allotted into three treatments: CON (basal diet, control), LFD and HFD (basal diet with 500 and 1000 mg/kg FD, respectively), with five replicates of eight birds each. The experiment lasted for 42 days.

**Results:**

The results showed that birds in HFD group had increased ADG during 1–21 days (*P* < 0.05). On day 21, the bursa of Fabricius index of birds in LFD group was higher *(P* < 0.05), while the serum contents of IFN-γ and TNF-ɑ were lower in HFD group (*P* < 0.05). FD supplementation decreased the observed_species, shannon, chao1 and ace indexes (*P* < 0.05) as well as the abundance of Bacteroidota, *Bacteroides*, and *Alistipes* (*P* < 0.05). Birds in HFD group had higher abundance of Firmicutes and lower abundance of Verrucomicrobiota (*P* < 0.05). LFD group had lower abundance of unidentified_bacteria (*P* < 0.05). On day 42, the abdominal fat yield of HFD group was decreased (*P* < 0.05). Birds in LFD group had lower L* and b* values of breast muscle (*P* < 0.05), while higher spleen index. The CAT activities of breast muscle of FD groups were higher (*P* < 0.05).

**Conclusion:**

In summary, dietary FD supplementation at 1000 mg/kg improved production performance and immune function and modulated microbiota composition in ileum of broiler chickens. FD can be supplemented in the diet to enhance performance and health of broiler chickens, of which 1000 mg/kg FD is more effective.

## Background

Dandelion (Taraxacum mongolicum Hand.-Mazz.) is a herb belonging to the Asteraceae family and widely distributed in the warmer temperate regions of the Northern Hemisphere [1]. Dandelion contains various bioactive substances, such as polyphenols, flavonoids, polysaccharides, alkaloids, terpenoids and peptides [2], and is used as a kind of traditional Chinese medicine for its variety of health benefits, such as antioxidant, anti-inflammatory, and antibacterial activities [3, 4]. In our previous study, dietary supplementation of dandelion improved growth performance, enhanced intestinal immunity and modified intestinal microbiota of broilers [5], suggesting that dandelion could be a potential feed additive in poultry industry. It was shown that the bioactive substances of herbs are located in the cell walls, which limits the utilization of the bioactive substances by domestic animals [6].

The microbial fermentation (MF) is a technology to degrade or transform macromolecular substances in feed materials into nutrients that can be better digested and absorbed by domestic animals, which has been widely applied to the feed industry [7]. It has been reported that enzymes including cellulose, pectinase, and hemicellulose produced during MF could degrade or disrupt cell walls, thus enabling better release of bioactive compounds from herbs as well as higher absorption and utilization by animals [8]. In our previous study, we optimized the fermentation conditions for dandelion, and found that the flavonoids content in fermented dandelion (FD) was significantly higher than raw dandelion [9]. Moreover, FD showed superior antioxidant activity in vitro and in vivo [9, 10]. Therefore, the current study evaluated whether FD could have beneficial consequences on production performance, meat quality, immune function, and intestinal microbiota of broiler chickens.

## Results

### Production performance

The effects of dietary FD supplementation on growth performance and carcass traits of broilers are presented in Tables 1 and 2, respectively. During day 1–21, the average daily gain (ADG) of birds in HFD group was increased by 12.22% compared with the CON group (*P* < 0.05). The feed to gain ratio (F/G) of birds in HFD group tended to be lower than that of LFD group (*P* = 0.063). The growth performance of broilers during 22–42 d and 1–42 d was not affected by FD supplementation. Furthermore, the dressed yield of birds in HFD group tended to be higher than that of LFD group (*P* = 0.088). Birds in HFD group had lowered the abdominal fat yield (27.94%) than CON group (*P* < 0.05).

**Table 1:** Effects of dietary FD supplementation on growth performance of broilers

Item	CON	LFD	HFD	SEM	*P*-value
1–21 d					
BW21	723.13	723.84	700.86	15.796	0.816
ADG	31.92^b^	31.31^b^	35.82^a^	0.834	0.033
ADFI	47.64	47.29	51.71	1.011	0.123
F/G	1.50^xy^	1.51^x^	1.44^y^	0.013	0.063
22–42 d					
BW42	2366.98	2288.53	2290.03	41.971	0.715
ADG	78.43	78.96	77.72	1.469	0.950
ADFI	144.38	133.58	143.56	2.907	0.255
F/G	1.84	1.69	1.86	0.039	0.163
1–42 d					
ADG	55.64	54.97	56.50	0.854	0.791
ADFI	96.14	90.43	92.74	1.992	0.535
F/G	1.73	1.67	1.64	0.028	0.417

*CON* Basal diet, *LFD*, Basal diet + 500 mg/kg FD, *HFD* Basal diet + 1000 mg/kg FD, *ADG* Average daily gain, *ADFI* Average daily feed intake, *F/G* Feed to gain ratio, *SEM* Standard error of the mean

^a,b^Means (*n* = 5) in a row with different letters differed significantly (*P* < 0.05)

^x,y^Means (*n* = 5) in a row with different letters tended to be different (0.05 ≤ *P* < 0.10)

**Table 2:** Effects of dietary FD supplementation on carcass traits of broilers

Item (%)	CON	LFD	HFD	SEM	*P*-value
Dressed yield	91.34^xy^	1. 91.02^y^	92.21^x^	0.233	0.088
Eviscerated yield	73.55	74.78	74.65	0.804	0.816
Abdominal fat yield	2.04^a^	1.92^ab^	1.47^b^	0.105	0.040
Breast muscle yield	19.21	18.99	20.32	0.451	0.438
Thigh muscle yield	26.28	27.32	26.93	0.438	0.673

*CON* Basal diet, *LFD* Basal diet + 500 mg/kg FD, *HFD* Basal diet + 1000 mg/kg FD, *SEM* Standard error of the mean

^a,b^Means (*n* = 5) in a row with different letters differed significantly (*P* < 0.05)

^x,y^Means (*n* = 5) in a row with different letters tended to be different (0.05 ≤ *P* < 0.10)

## Meat quality

The results regarding the effects of dietary FD supplementation on physical characteristics and antioxidant indexes of breast muscle are presented in Tables 3 and 4. As showed in Table 3, the lightness (L*) and yellowness (b*) of birds in LFD group were significantly lower than that of the CON group (*P* < 0.05). In addition, compared with the CON group, the drip loss of birds in LFD group showed a tendency to be lower than CON group (*P* = 0.088). Compared with CON group, the catalase (CAT) activities of breast muscle of birds in FD groups were increased (*P* < 0.05), and the total superoxide dismutase (T-SOD) activity of LFD group tended to be higher (*P* = 0.053).

**Table 3:** Effects of dietary FD supplementation on physical characteristics of breast muscle in broilers

Item	CON	LFD	HFD	SEM	*P*-value
pH 0.75	6.56	6.54	6.54	0.055	0.987
pH 24	6.03	6.09	6.10	0.047	0.843
Lightness (L*)	47.64^a^	45.31^b^	46.27^ab^	0.387	0.032
Redness (a*)	2.55	2.75	2.58	0.163	0.868
Yellowness (b*)	10.58^a^	9.33^b^	10.03^ab^	0.207	0.020
Water loss, %	17.18	19.13	16.64	1.056	0.648
Drip loss, %	3.07^x^	2.34^y^	2.60^xy^	0.146	0.088
Cooking loss, %	31.56	26.58	26.34	1.291	0.181
Shear force, N	60.91	50.48	58.84	3.380	0.436

*CON* Basal diet, *LFD* Basal diet + 500 mg/kg FD, *HFD* Basal diet + 1000 mg/kg FD, *SEM* Standard error of the mean

^a,b^Means (*n* = 5) in a row with different letters differed significantly (*P* < 0.05)

^x,y^Means (*n* = 5) in a row with different letters tended to be different (0.05 ≤ *P* < 0.10)

**Table 4:** Effects of dietary FD supplementation on antioxidant indexes of breast muscle in broilers

Item	CON	LFD	HFD	SEM	*P*-value
T-SOD, U/mg pro	520.37^y^	661.52^x^	530.11^y^	28.770	0.053
CAT, U/mg pro	14.66^b^	38.61^a^	33.83^a^	3.270	0.001
GSH-PX, U/mg pro	11.53	17.15	16.63	1.433	0.206
GSH, μmol/g pro	133.02	172.03	182.63	15.180	0.380
MDA, μmol/g pro	3.82	1.77	2.64	0.400	0.102

*CON* Basal diet, *LFD* Basal diet + 500 mg/kg FD, *HFD* Basal diet + 1000 mg/kg FD, *SEM* Standard error of the mean

^a,b^Means (*n* = 5) in a row with different letters differed significantly (*P* < 0.05)

^x,y^Means (*n* = 5) in a row with different letters tended to be different (0.05 ≤ *P* < 0.10)

### Immune function

Tables 5 and 6 show the immune function of broilers fed with FD, including immune organ indexes and serum inflammatory cytokines contents. Compared with the CON group, the Bursa of Fabricius index on day 21 and spleen index on day 42 of birds in LFD group were higher (*P* < 0.05). As showed in Table 6, the serum interferon-γ (IFN-γ) and tumor necrosis factor-α (TNF-ɑ) contents of HFD group was lower than that of the CON group on day 21 (*P* < 0.05). In addition, compared with the CON group, the serum interleukin-8 (IL-8) content of HFD group showed a tendency to be decreased on day 42 (*P* = 0.090).

**Table 5:** Effects of dietary FD supplementation on immune organ indexes of broilers

Item	CON	LFD	HFD	SEM	*P*-value
21 d					
Bursa of Fabricius index	0.19^b^	0.29^a^	0.23^ab^	0.016	0.025
Spleen index	0.09	0.09	0.10	0.004	0.263
Thymus index	0.46	0.50	0.42	0.025	0.417
42 d					
Bursa of Fabricius index	0.15	0.13	0.16	0.013	0.730
Spleen index	0.08^b^	0.13^a^	0.10^ab^	0.008	0.021
Thymus index	0.42	0.43	0.50	0.020	0.168

*CON* Basal diet, *LFD* Basal diet + 500 mg/kg FD, *HFD* Basal diet + 1000 mg/kg FD, *SEM* Standard error of the mean

^a,b^Means (*n* = 5) in a row with different letters differed significantly (*P* < 0.05)

**Table 6:** Effects of dietary FD supplementation on serum inflammatory cytokines contents of broilers

Item(pg/ml)	CON	LFD	HFD	SEM	*P*-value
21 d					
IFN-γ	228.68^a^	206.90^ab^	190.70^b^	5.999	0.023
IL-8	221.98	212.97	200.33	6.108	0.370
IL-10	83.78	75.87	81.48	3.314	0.637
IL-1β	103.31	102.96	93.50	2.882	0.306
TNF-ɑ	100.92^a^	93.85^a^	81.12^b^	2.918	0.009
42 d					
IFN-γ	188.28	192.25	180.98	3.673	0.500
IL-8	189.95^x^	186.00^xy^	163.71^y^	5.181	0.090
IL-10	71.14	71.73	71.64	4.081	0.998
IL-1β	99.44	81.13	80.34	5.302	0.255
TNF-ɑ	98.68	90.94	86.70	3.105	0.302

*CON* Basal diet, *LFD* Basal diet + 500 mg/kg FD, *HFD* Basal diet + 1000 mg/kg FD, *SEM* Standard error of the mean

^a,b^Means (*n* = 5) in a row with different letters differed significantly (*P* < 0.05)

^x,y^Means (*n* = 5) in a row with different letters tended to be different (0.05 ≤ *P* < 0.10)

### Intestinal microbiota

**Fig. 1:**
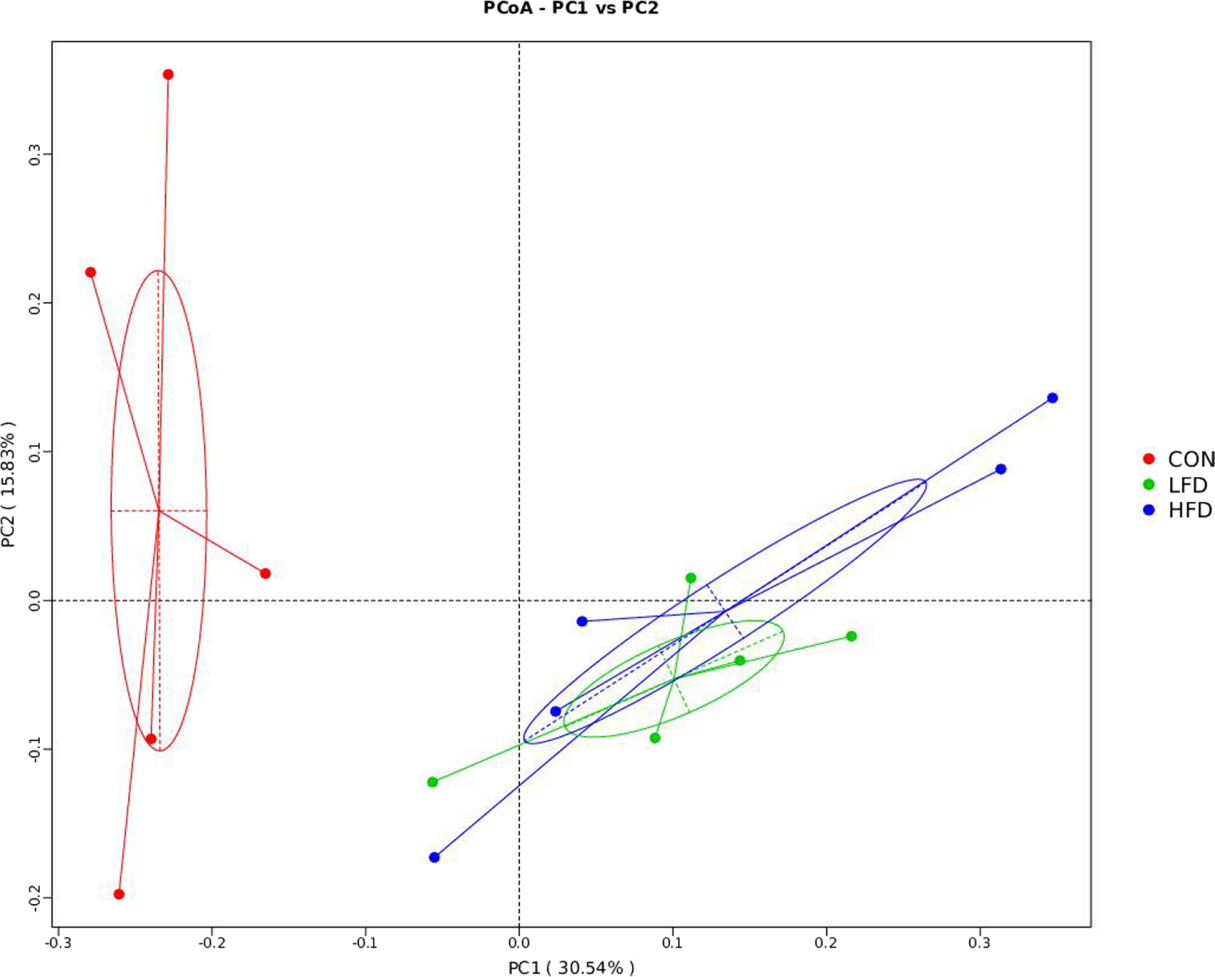
Effects of dietary FD supplementation on ileal microbial β-diversity of broilers on d 21. CON = basal diet; LFD = basal diet + 500 mg/kg FD; HFD = basal diet + 1000 mg/kg FD

**Fig. 2:**
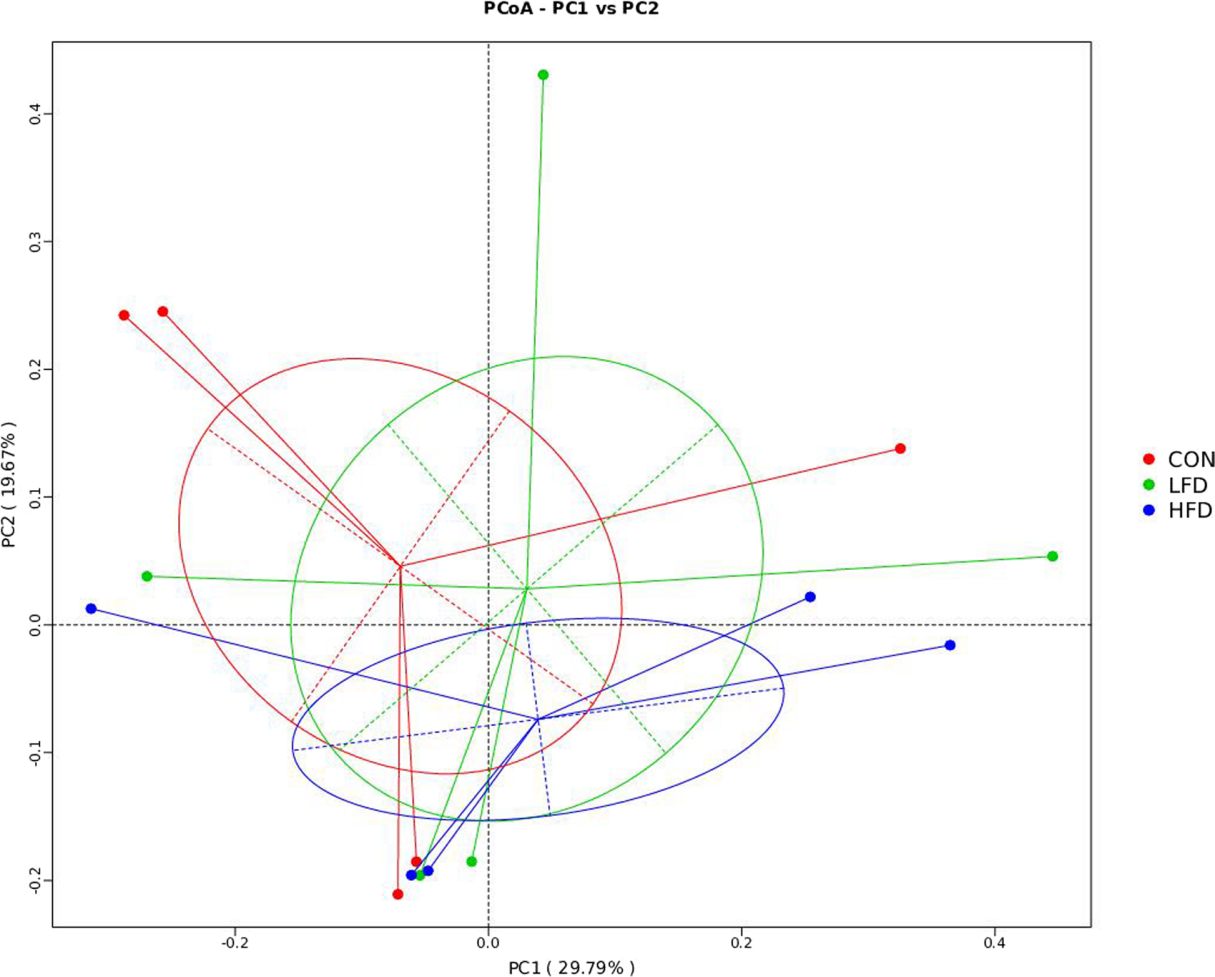
Effects of dietary FD supplementation on ileal microbial β-diversity of broilers on d 42. CON = basal diet; LFD = basal diet + 500 mg/kg FD; HFD = basal diet + 1000 mg/kg FD

Table 7 displays the α-diversity in ileal microbiota of broilers fed with FD, including observed_species, shannon, chao1, and ace indices. On day 21, birds in FD supplemented groups had decreased observed_species, shannon, chao1, and ace indices in comparison with the CON group (*P* < 0.05). On day 42, no significant difference in alpha diversity indices was found among all groups (*P* > 0.05). Figures 1 and 2 show the effects of dietary FD supplementation on ileal microbial β-diversity of broilers. The Principal coordinate analysis (PCoA) based on unweighted_unifrac distance metrics revealed statistically significant discrimination between the FD supplemented groups and the CON group (PC1, 30.54%; PC2, 15.83%) on day 21. However, there were no significant differences for the bacterial communities among FD and CON groups on day 42.

**Table 7:** Effects of dietary FD supplementation on alpha diversity of ileal microbiota in broilers

Item	CON	LFD	HFD	SEM	*P*-value
21 d					
observed_species	310^a^	148^b^	140^b^	25.708	0.009
shannon	4.09^a^	2.50^b^	2.37^b^	0.264	0.013
chao1	333.87^a^	168.16^b^	155.25^b^	26.081	0.009
ace	338.67^a^	174.71^b^	162.77^b^	25.865	0.009
42 d					
observed_species	505	512	517	40.254	1.000
shannon	3.45	3.57	3.67	0.336	0.961
chao1	558.50	579.00	562.40	42.355	0.970
ace	567.80	595.40	574.30	42.377	0.970

*CON* Basal diet, *LFD* Basal diet + 500 mg/kg FD, *HFD* Basal diet + 1000 mg/kg FD, *SEM* Standard error of the mean

^a,b^Means (*n* = 5) in a row with different letters differed significantly (*P* < 0.05)

^x,y^Means (*n* = 5) in a row with different letters tended to be different (0.05 ≤ *P* < 0.10)

**Fig. 3:**
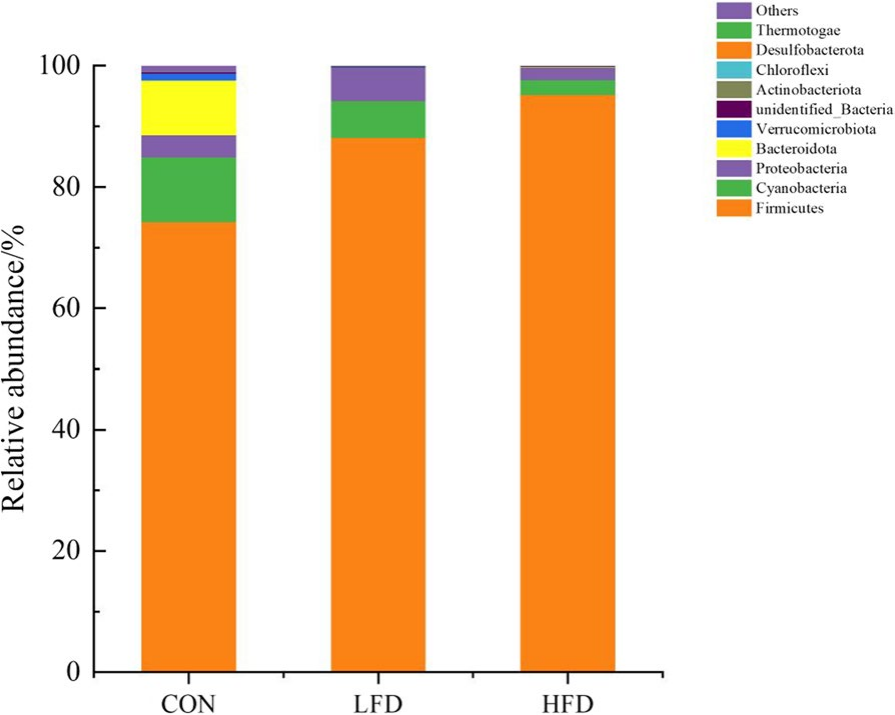
Effects of dietary FD supplementation on the relative abundances of ileal microbiota at the phyla level (21 days old). Only bacteria with the top 10 relative abundance are shown in the Figure. CON = basal diet; LFD = basal diet + 500 mg/kg FD; HFD = basal diet + 1000 mg/kg FD

**Fig. 4:**
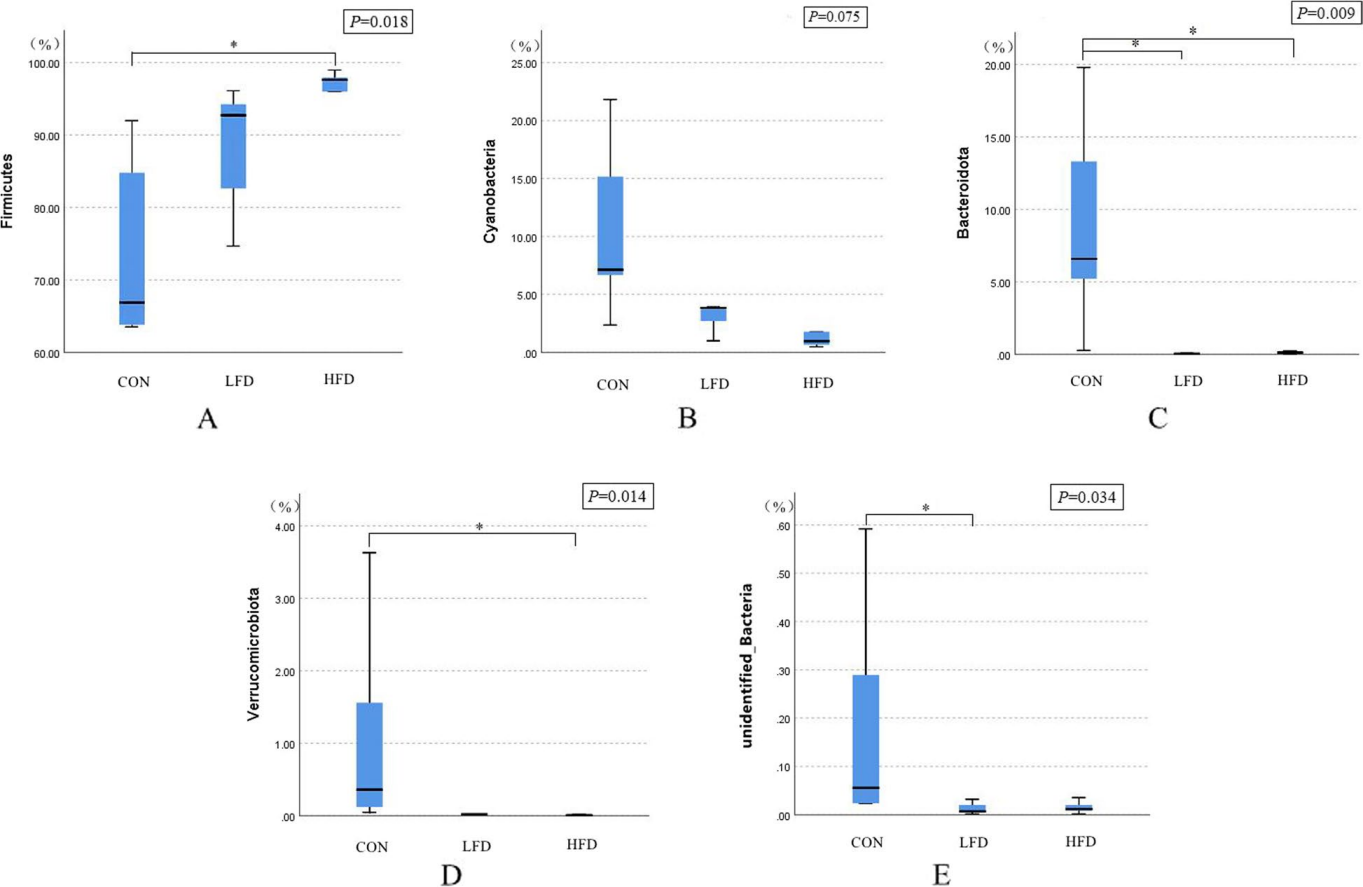
Effects of dietary FD supplementation on the relative abundances of Firmicutes (**A**), Cyanobacteria (**B**), Bacteroidota (**C**), Verrucomicrobiota (**D**), and unidentified_Bacteria (**E**) (21 days old). *Means (*n* = 5) two groups differed significantly (*P* < 0.05). CON = basal diet; LFD = basal diet + 500 mg/kg FD; HFD = basal diet + 1000 mg/kg FD

**Fig. 5:**
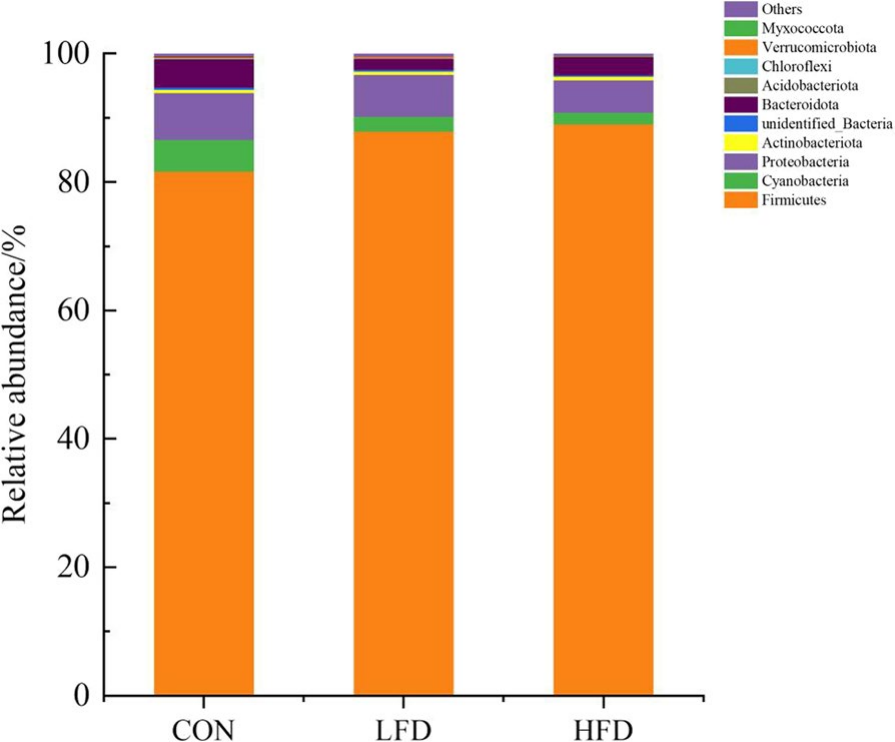
Effects of dietary FD supplementation on the relative abundances of ileal microbiota at the phyla level (42 days old). Only bacteria with the top 10 relative abundance are shown in the Figure. CON = basal diet; LFD = basal diet + 500 mg/kg FD; HFD = basal diet + 1000 mg/kg FD

The effects of dietary supplementation with FD on the relative abundances of ileal microbiota at the phyla level are presented in Figs. 3, 4 and 5. As shown in Figs. 3 and 5, Firmicutes was the dominant phylum in ileal microbiota of broilers on day 21 and 42. As shown in Fig. 4, compared with CON group, FD supplemented groups had lower relative abundances of Cyanobacteria (*P* = 0.075) and Bacteroidota (*P* = 0.009) on day 21.The relative abundances of unidentified_Bacteria of birds in LFD group was lower than CON group (*P* < 0.05). In addition, birds in HFD group had higher relative abundances of Firmicutes and lower relative abundances of Verrucomicrobiota compared with CON group (*P* < 0.05). There was no significant difference in the relative abundance of ileal bacteria between FD supplemented groups and CON group on day 42.

**Fig. 6:**
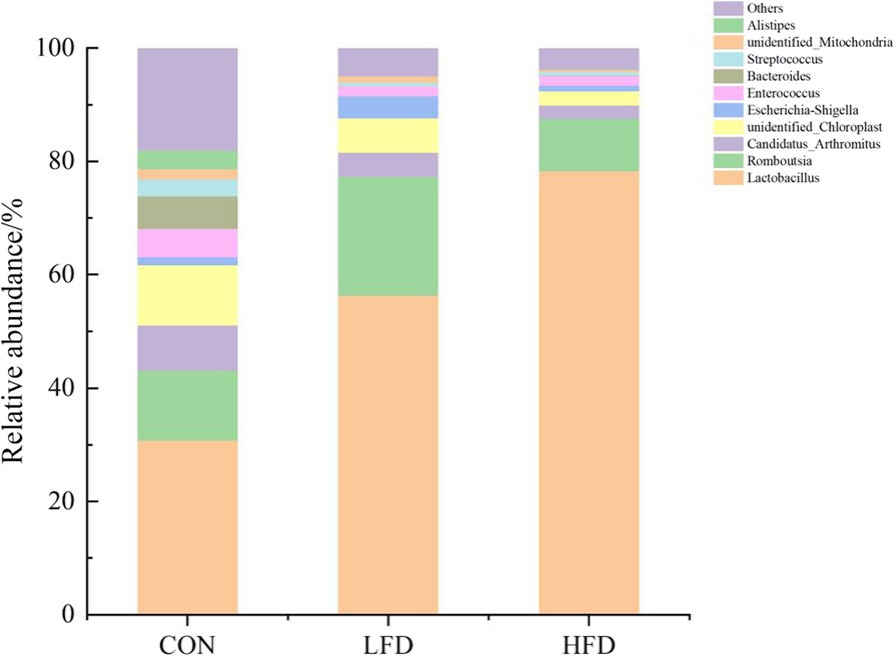
Effects of dietary FD supplementation on the relative abundances of ileal microbiota at the genus level (21 days old). Only bacteria with the top 10 relative abundance are shown in the Figure. CON = basal diet; LFD = basal diet + 500 mg/kg FD; HFD = basal diet + 1000 mg/kg FD

**Fig. 7:**
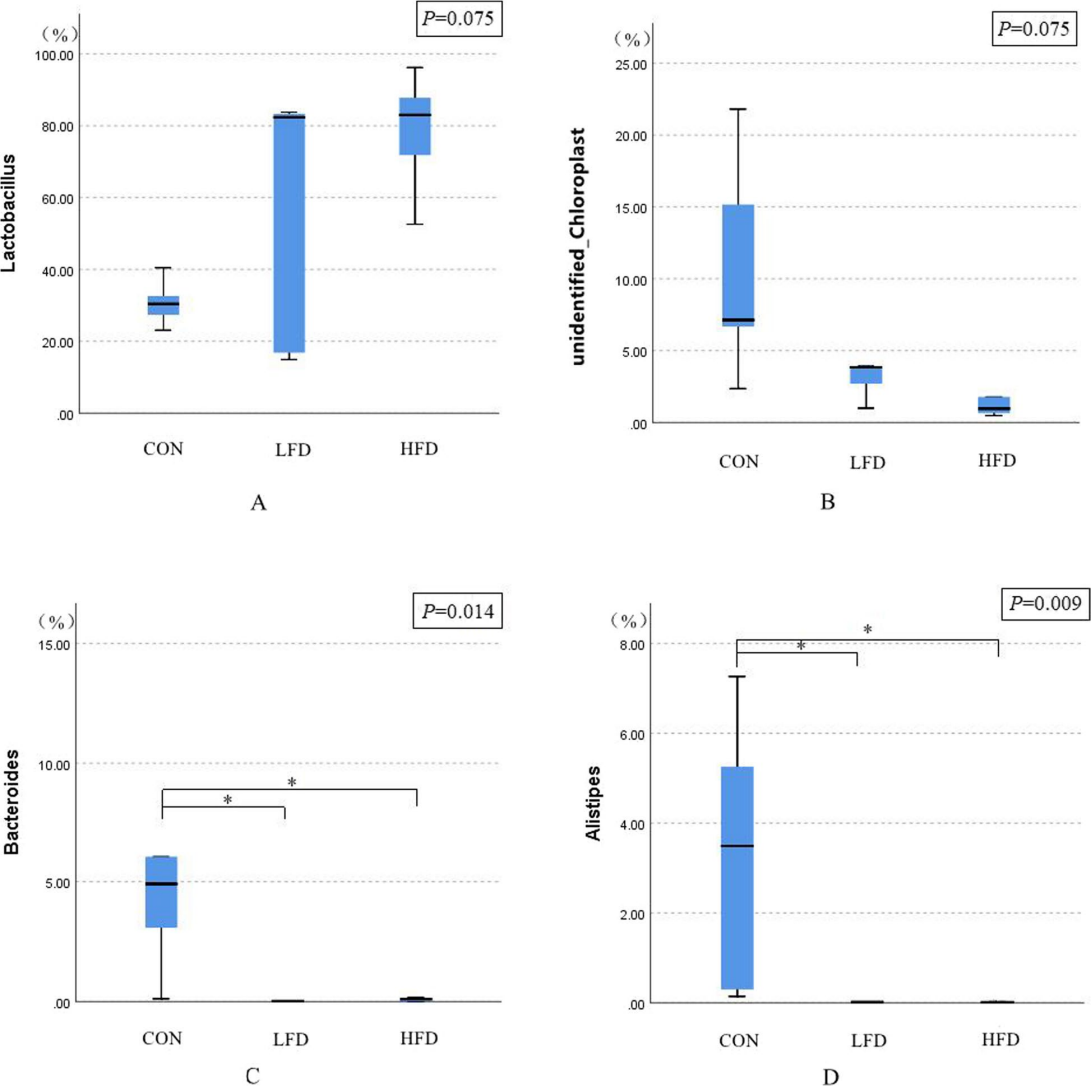
Effects of dietary FD supplementation on the relative abundances of *Lactobacillius* (**A**), *unidentified_Chloroplast* (**B**), *Bacteroides*(**C**) and *Alistipes* (**D**) (21 days old). *Means (*n* = 5) two groups differed significantly (*P* < 0.05). CON = basal diet; LFD = basal diet + 500 mg/kg FD; HFD = basal diet + 1000 mg/kg FD

**Fig. 8:**
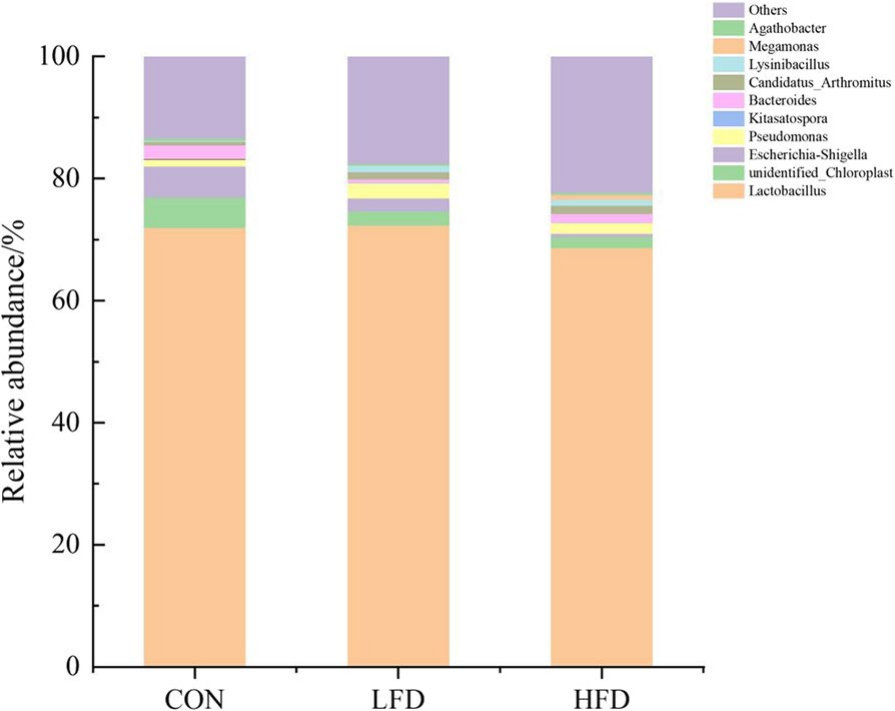
Effects of dietary FD supplementation on the relative abundances of ileal microbiota at the genus level (42 days old). Only bacteria with the top 10 relative abundance are shown in the Figure. CON = basal diet; LFD = basal diet + 500 mg/kg FD; HFD = basal diet + 1000 mg/kg FD

**Fig. 9:**
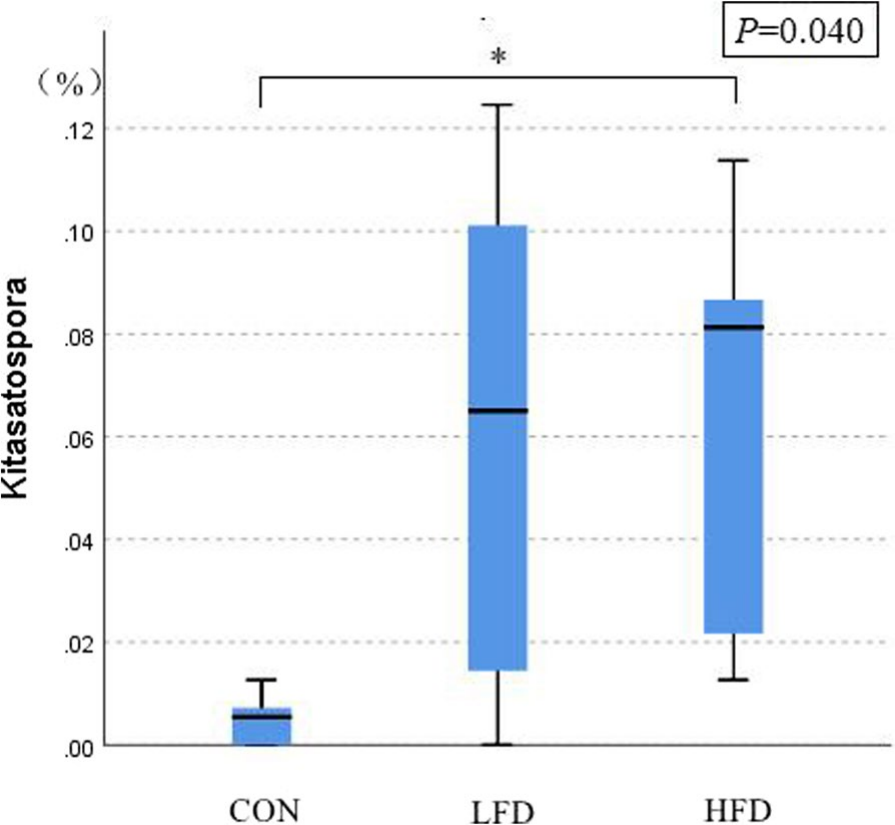
Effects of dietary FD supplementation on the relative abundances of *Kitasatospora* (42 days old). *Means (*n* = 5) two groups differed significantly (*P* < 0.05). CON = basal diet; LFD = basal diet + 500 mg/kg FD; HFD = basal diet + 1000 mg/kg FD

The effects of dietary supplementation with FD on the relative abundances of ileal microbiota at the genera level are presented in Figs. 6, 7, 8 and 9. As shown in Figs. 6 and 8, *Lactobacillus* was the dominant genus in the ileal microbiota, which belonging to Firmicutes. As shown in Fig. 7, compared with CON group, birds in FD supplemented groups had higher relative abundances of *Lactobacillus* (*P* = 0.075) and lower relative abundances of *unidentified_Chloroplast* (*P* = 0.075), *Bacteroides* (*P* = 0.014) and *Alistipes* (*P* = 0.009) on day 21. On day 42, compared with CON group, birds in HFD group had significantly higher relative abundances of *Kitasatospora* (*P* = 0.040) (Fig. 9).

## Discussion

Asteraceae plants have already been studied due to their abundant bioactive substances, which are conducive to improve the performance of poultry [11–13]. Previous results revealed that dietary supplementation with 1 g/kg dandelion leaf powder increased the total weight gain for 1–21 day and 1–35 day, and decreased the cumulative feed conversion factor for 1–21 day [14]. Yan et al. (2012) [15] found that dietary inclusion with 1 g/kg dandelion increased the ADG and the G:F ratio. However, Oh et al. (2007) [16] showed that dietary 1.0% dandelion supplementation had no effect on growth performance of broilers. It may be due to that the bioactive substances of dandelion are located in the cell walls, which limits the utilization of the bioactive substances by domestic animals. In current study, we found that dietary inclusion of 1000 mg/kg FD increased the ADG and tended to decrease F/G of broilers. It is possible that the bioactive compounds, which played a crucial role in promoting growth and stimulating metabolic of broilers [16], were released by MF. It was confirmed that the contents of flavonoids, polyphenols, and polysaccharides of FD used in present study were 48.84, 56.82, and 110.42 mg/g, respectively. Compared with raw dandelion, the contents of flavonoids and polyphenols were increased by 39.54% and 30.68%. It is also reported that the utilization rate of bioactive substances in herbs was enhanced after fermentation [8]. In addition, in the present study, bird in HFD group had higher dressed yield and lower abdominal fat yield. Similarly, Zhou et al. (2022) [17] indicated that dandelion increased the dressed yield by reducing the abdominal fat yield of broilers. It may be due to that flavonoids in dandelion can inhibit fat deposition in broilers [17].

Chicken meat is one of the major animal protein around the world due to its desirable nutritive value, and more and more customers pay attention to the quality of chicken meat [18, 19]. Generally, meat quality is reflected by some indexes, such as pH, meat color, water loss, cooking loss, drip loss, shear force and so on [20]. Meat color is reflected by L*, redness (a*) and b* values, in which L* is related to the water-holding capacities (WHC) and a* and b* are linked to antioxidant indexes [21–23]. Previous studies demonstrated the positive effects of Asteraceae plants and their bioactive compounds on the meat quality of poultry [24, 25]. Jin et al. (2021) [26] showed that 500 mg/kg of resveratrol, a polyphenol, reduced the values of L* and b* of duck breast muscle. However, there are only a few literature reports on the effect of dandelion on meat quality in broilers. In current study, 500 mg/kg FD decreased the L*, b* and drip loss of breast muscle in broilers. Similarly, Du et al. (2022) [6] found that inclusion of 1 g/kg enzymatically treated dandelion reduced drip loss of breast muscle, while raw dandelion had no effects. It is possible that the polyphenols and flavonoids released by MF or enzymatically treatment exhibited free radicals scavenging activity, which could be due to their electron or hydrogen providing property [27]. The research of Liu et al. (2020) [9] and Yin et al. (2022) [10] confirmed this view. Moreover, the beneficial effects of FD on physical characteristics of breast muscle could be related to the improved antioxidant indexes [28, 29], which was confirmed by the increased T-SOD and CAT activities in current study. T-SOD and CAT are important antioxidant enzymes, which are responsible for removing free radicals and improving antioxidant status of animals. However, our study found no effect of 1000 mg/kg FD on physical characteristics of breast muscle, while with increased CAT activity. The discrepancy in the physical characteristics of meat need further research.

It has been proven that dietary supplementation with Asteraceae plants and their extracts can improve immune function of animal [30–32]. The immune organ indexes are crucial for assessing the condition of immune function and the growth of animal [33]. The bursa of Fabricius is in charge of the maturation of B-lymphocytes, and the spleen plays a role in regulating cellular and humoral immunity through the activation of T-lymphocytes, B-lymphocytes, and macrophage [34]. Yang et al. (2013) [35] found that dietary supplementation with 0.3% and 0.5% Taraxacum polysaccharides increased the spleen and thymus indices. In line with previous study, our study showed that birds fed diets with 500 mg/kg FD exhibited higher bursa of Fabricius index on day 21 and higher spleen index on day 42. The growth and development of immune organs may attribute to the polysaccharide of dandelion [35]. In addition, cytokines, a type of protein that induced by immunogens or other exogenous stimulants, contribute cell-mediated immune response [36, 37]. Previous research found that polysaccharides of Atractylodes macrocephala Koidz, belonged to Asteraceae family, decreased the IFN-γ and TNF-α levels in chicken immune organs [38]. Consistent with this result, our study found that 1000 mg/kg FD decreased the serum IFN-γ, TNF-α and IL-8 contents of broilers. The IFN-γ, TNF-α and IL-8 are important mediators of the inflammatory response, which play key roles when inflammation occurs [39, 40]. These results imply that dietary supplementation with 1000 mg/kg FD could reduce the likelihood of inflammation in broilers. Furthermore, these results confirm that FD (1000 mg/kg) could promote production performance of broilers by decreasing related energy expenditures in immune system.

Intestinal microbiota shows strong relation with digestion and absorption of nutrients and immune response of host [41]. A number of studies have shown that Asteraceae plants modified intestinal microbiota of poultry [42–44]. However, few studies have been conducted to examine the effects of dandelion on the intestinal microbiota of poultry. Tan et al. (2020) [45] showed that the shannon, chao1, and ace indices of gut microbiota in golden pompano was increased by 1 g/kg dandelion extract. However, our previous study conducted with broilers found that dandelion decreased the α-diversity of ileal microbiota on day 21 [5]. Similarly, in this study, we found that birds fed FD had decreased observed_species, shannon, chao1, and ace indices on day 21. It is suggested that the reduction of the richness and diversity of ileal microbiota may be related to the antibacterial activity of dandelion [46]. In addition, the result of β-diversity showed that the intestinal microbiota of birds in LFD and HFD groups was different than that in CON group, which may also be due to the antibacterial activity of dandelion [46]. Firmicutes, the key strain producing short chain fatty acids (SCFAs), can provide intestinal cells with more effective energy source and enhance carbohydrate absorption of host [47]. It is reported that ADG of animal was positively associated with Firmicutes abundance and negatively associated with Bacteroidota abundance [48]. Therefore, the relative abundance of Firmicutes and Bacteroidota could be considered as a biomarker to analyze the growth performance of animal. *Lactobacillius*, a probiotics, is advantageous to the synthesis of lactate and SCFAs, which can protect the integrity of the intestinal mucosal, hinder the proliferation of pathogenic microbe, and stimulate the immune system [24, 49]. In addition, *Kitasatospora* has been found to produce at least 50 bioactive compounds, which can inhibit pathogenic bacteria and reduce host inflammation, thus producing a certain growth-promoting effect [50]*.* In this study, 1000 mg/kg FD increased the relative abundances of Firmicutes and *Lactobacillius* on day 21, decreased the relative abundances of Bacteroidota and *Bacteroides* on day 21, and increased the relative abundances of *Kitasatospora* on day 42. Similar to our results, previous studies showed that fermented herbs supplementation increased the Firmicutes to Bacteroidota ratio and the relative abundance of *Lactobacillius* in piglets [51]. This may be the reason for the improved ADG and reduced inflammatory response of broilers fed with 1000 mg/kg FD in this research. Additionally, the relative abundance of Cyanobacteria was lower in FD supplemented groups on day 21, which was consistent with Chen et al. (2021) [52]. Cyanobacteria, a harmful bacterium, exhibit potential neurotoxic or proinflammatory activities [52]. The reduction of Cyanobacteria relative abundance reflected that the herbs are effective against pathogenic bacteria [53]. In our current study, the relative abundance of Verrucomicrobiota were lower in FD supplemented groups on day 21. However, there has been limited research showing the effect of Verrucomimicrobiota on broiler intestines at present; thus, it is difficult to give any direct explanation.

## Conclusions

Supplementation of FD at 1000 mg/kg improved ADG, decreased abdominal fat yield and serum proinflammatory cytokines (IFN-γ, TNF-ɑ and IL-8) contents, enhanced antioxidative enzymes (T-SOD and CAT) activities of breast muscle and modified intestinal microbiota by increasing the abundance of Firmicutes and Lactobacillius and decreasing the abundance of Bacteroidota, Bacteroides, and Cyanobacteria. Meanwhile, 500 mg/kg FD supplementation reduced L* and b* values and shear force of breast muscle. In summary, FD can be supplemented in the diet to enhance performance and health of broiler chickens, of which 1000 mg/kg FD is more effective.

## Methods

### Ethics statement

The study was approved by Inner Mongolia Agricultural University Research Ethics Committee (permission number of [2020]065). The study was carried out in accordance with relevant guidelines and regulations (GB 14925–2010). All the methods are reported in accordance with ARRIVE guidelines.

### The fermentation of dandelion

According to the previous research of our laboratory [10], the compound probiotics include *Saccharomyces cerevisiae* (1.0 × 10^8^ CFU/mL) and *Lactobacillus Plantarum* (1.0 × 10^8^ CFU/mL) at the ration of 7:3. The raw dandelion was inoculated with 10% (v/v) the compound probiotics and 0.5% yeast extract. Distilled water was added a ratio of 1:1.2. Then fermentation was conducted at 37℃ for 50 h. After fermentation, the FD was obtained by drying at 45℃ and grounding with a hammer mill. Then, the content of flavonoids, polyphenols, and polysaccharides in raw dandelion power and FD power was determined. Referring to Liu et al. [9], the colorimetric method was used to determine the total flavonoid content using rutin as a standard substance. Determination of polyphenols and polysaccharides was conducted by folin phenol method [54] and phenol–sulfuric acid method [55], respectively. The flavonoids, polyphenols, and polysaccharides contents of raw dandelion were 35.00, 43.48, and 193.93 mg/g, respectively. Then the contents of the corresponding bioactive substances of FD were 48.84, 56.82, and 110.42 mg/g, respectively. 

### Animal, diet, and experiment design

A total of 120 healthy 1-day-old male Arbor Acres broilers were purchased from Inner Mongolia Yuean Breeding Co., Ltd. The broilers were randomly divided into three dietary treatments: (1) CON (control, corn-soybean meal basal diet without additives), (2) LFD (basal diet with 500 mg/kg FD), and (3) HFD (basal diet with 1000 mg/kg FD). In each group, there were five replicates with eight chickens per replicate. The experiment lasted for 42 days, including a starter phase (d 1 to 21) and a grower phase (d 22 to 42). The basal diets were made in accordance with the nutrient recommendations of Feeding Standard of Chicken, China (NY/T 33–2004) (Chinese Ministry of Agriculture, 2004). The nutrient levels including crude protein (CP), ether extract (EE), crude fiber (CF), calcium, and phosphorus were determined according to the Association of the AOAC (2000) [56]. Dietary amino acid concentrations were analyzed using HPLC (L-8900 AA Analyzer, Hitachi, Tokyo, Japan) as previously described by Heinrikson et al. (1984) [57]. The compositions and nutrient levels of the basal diets for starter and grower phases are listed in Table 8.

**Table 8:** Composition and nutrient levels of basal diets

Items	Starter phase (d 1 to 21)	Grower phase (d 22 to 42)
Ingredient (%)		
Corn	52.50	58.80
Soybean meal	40.00	33.80
Soybean oil	3.00	3.00
Dicalcium phosphate	1.90	1.80
Limestone	1.08	1.22
Salt	0.37	0.37
Lysine	0.05	0.03
Methionine	0.19	0.07
Premix^a^	0.80	0.80
Choline	0.11	0.11
Total	100.00	100.00
Nutrient levels^b^		
Metabolic energy (MJ/kg)	12.42	12.62
Crude protein (%)	22.27	19.24
Ether extract (%)	4.69	5.45
Crude fiber (%)	3.50	3.26
Calcium (%)	1.17	1.18
Total phosphorus (%)	0.82	0.77
Non-phytate phosphorus (%)	0.53	0.49
Lysine (%)	1.39	1.22
Methionine (%)	0.35	0.32
Threonine (%)	0.90	0.80
Tryptophan (%)	0.78	0.70

^a^The premix provided the following per kg of diet: VA, 9000 IU; VD_3_, 3000 IU; VE, 26 mg; VK_3_, 1.20 mg; VB_1_, 3.00 mg; VB_2_, 8.00 mg; VB_6_, 4.40 mg; VB_12_, 0.012 mg; nicotinic acid, 45 mg; folic acid, 0.75 mg; biotin, 0.20 mg; choline, 1100 mg; D-pantothenic acid, 15 mg; Fe, 100 mg; Cu, 10 mg; Zn, 108 mg; Mn, 120 mg; I 1.5 mg; Se, 0.35 mg

^b^Metabolic energy is a calculated value, while the others are measured values

During the whole experiment, the chickens were reared in stainless-steel wire cages and had free access to feed and water. The room temperature was kept at 35℃ for the first week, then gradually decreased by 2–3℃ per week until the final temperature reached 25℃. The lighting schedule was continuous light on day 1–3, followed by 16 h of lighting and 8 h of darkness up to day 14, then natural light until the end of the trial.

### Growth performance and carcass traits

Body weight (BW) of each bird and feed intake of each replicate were weighed and recorded on day 21 and day 42, which were used to calculate the average daily gain (ADG), average daily feed intake (ADFI), and feed to gain ratio (F/G) for starter, grower and overall periods.

On day 42 of the experiment, one bird per replication (5 per treatment) was randomly selected to be slaughtered after 12 h fasting. In the experiment, the slaughter trial used euthanasia for cervical dislocation. The abdominal fat, breast muscle and thigh muscle were removed and weighted. Dressed yield and eviscerated yield were calculated as a percentage of BW. Abdominal fat yield was calculated as abdominal fat weight / (abdominal fat weight + eviscerated weight). Breast muscle yield and thigh muscle yield were calculated as a percentage of eviscerated yield.

### Meat quality

The separated breast muscle was divided into different sizes and used for the determination of pH 45 min (pH0.75) and pH 24 h (pH24), meat color, water loss, drip loss, cooking loss and shear force. A subsample of breast muscle was quick-frozen in liquid nitrogen for antioxidant indexes analysis.

The pH values were measured at 45 min and 24 h postmortem using the portable pH meter (Corning Glass Works, Medfield, MA) [58]. Meat color, including L* (lightness), a* (redness) and b* (yellowness), were evaluated by the Minolta color-guide (BYK-Gardener GmbH, Geretsried, Germany) [14]. Water loss was measured by weight before and after 35-kg force for 5 min using a pressure instrument (RH-1000, Guangzhou Runhu Instrument Co., LTD, China) [58]. Drip loss was expressed in percentage as the weight difference before and after hanging [59]. Briefly, the breast muscle was trimmed to 5 × 2 × 1 cm size, weighed, hung at 4℃ for 24 h, and then gently dapped dry with a paper towel and reweighed. Cooking loss was determined as the weight difference between the initial raw and final cooked samples that were heated in an 85℃ water bath for 15 min [60]. Shear force was detected by slicing cooked samples into long strips (3 cm × 1 cm × 1 cm) along the direction parallel to the muscle fiber and shearing perpendicular to the muscle fiber using a texture analyzer (C-LM2, Xieli Technology Development Co., LTD, Qinhuangdao, China) [61].

The subsamples of breast muscle were homogenized according to Wang et al. (2021) [62]. The colorimetric kits (Nanjing Jiancheng Institute of Bioengineering) were used to assay the activities of total superoxide dismutase (T-SOD), glutathione peroxidase (GSH-Px), and catalase (CAT) and the content of malondialdehyde (MDA) and glutathione (GSH) in breast muscle.

### Immune function

On d 21 and d 42, one bird was randomly chosen from each replicate for sampling. Immune organs, including thymus, spleen, and bursa of fabricius, were collected and weighted postmortem. Then the immune organ indexes were calculated as the following equation:

$$\mathrm{Immune}\;\mathrm{organ}\;\mathrm{index}\;(\%)\:=\:\mathrm{immune}\;\mathrm{organ}\;\mathrm{weighted}\;(\mathrm g)/\mathrm{BW}\;(\mathrm g)\:\times\:100\%$$

Immune organ index (%) = immune organ weighted (g)/BW (g) × 100%

The blood was collected by jugular vein with vacuum blood collection needle. The blood was centrifuged at 3500 r/min for 10 min to obtain serum. The serum was stored at -80℃ for further inflammatory cytokines analysis. The contents of interferon-γ (IFN-γ), interleukin (IL)-8, IL-10, IL-1β, and tumor necrosis factor-α (TNF-α) were determined by commercial ELISA kits (Wuhan Jiyinmei biotechnology Co., Ltd., Hubei, China). The determination followed the kits’ instruction.

### Microbiota analysis

The ileum was removed from the birds which were slaughtered on day 21 and 42. The ileal digesta was compressed into a sterile tube, frozen in liquid nitrogen, and kept at -80℃ until further microbiota analysis. Bacterial 16S rRNA gene sequencing was used to analyze the ileal microbiota. The α-diversity, β-diversity, and the relative abundance of phyla and genus were analyzed referring to Mao et al. (2022) [5].

### Statistical analysis

Experiment data for growth performance, carcass traits, meat quality and immune function were analyzed by one-way ANOVA of SAS (SAS 9.2, SAS Institute Inc., Cary, NC), and means were separated by LSD multiple comparisons test. The replicate was considered as the experimental unit for growth performance and the bird for other data. Data were expressed as the means and standard error of the mean (SEM). Differences were considered significant at *P* < 0.05, and tendency when 0.05 ≤ *P* < 0.10.

For ileal microbiota profiling, kruskal–wallis test was used to analyze statistical significance of alpha diversity and bacterial community composition, and pairwise comparisons was used to conduct post-hoc multiple comparisons. Principal coordinate analysis (PCoA) was conducted to estimate beta-diversity. Differences were considered significant at *P* < 0.05, and tendency when 0.05 ≤ *P* < 0.10.

## Data Availability

The datasets used and/or analysed during the current study are available from the corresponding author on reasonable request. The original sequence data are available at the SRA by accession number PRJNA925308 (https://www.ncbi.nlm.nih.gov/sra/PRJNA925308).

## Abbreviations

MF: Microbial fermentation
FD: Fermented dandelion
CON: Basal diet, control
LFD: Basal diet with 500 mg/kg FD
HFD: Basal diet with 1000 mg/kg FD
BW: Body weight
ADG: Average daily gain
ADFI: Average daily feed intake
F/G: Feed to gain ratio
*L**: Lightness
*a**: Redness
*b**: Yellowness
T-SOD: Total superoxide dismutase
GSH-Px: Glutathione peroxidase
CAT: Catalase
MDA: Malondialdehyde
GSH: Glutathione
IL: Interleukin
IFN-γ: Interferon-γ
TNF-α: Tumor necrosis factor-α
PCoA: Principal coordinate analysis
CP: Crude protein
EE: Ether extract
CF: Crude fiber
